# Letter to the editor: efficacy of OLIF combined with pedicle screw internal fixation for lumbar spinal stenosis on spinal canal changes before and after surgery

**DOI:** 10.1186/s13018-023-04316-0

**Published:** 2023-11-16

**Authors:** Wangbing Xu, Faming Zhong, Weibing Liu

**Affiliations:** 1 Department of Spinal Orthopedics, Affiliated Hospital of Jiangxi University of Chinese Medicine, Nanchang, 330006 Jiangxi China; 2 Jiangxi University of Chinese Medicine, Nanchang, 330004 Jiangxi China

Dear Dr. Alimohammadi:

First and foremost, we extend our heartfelt appreciation to both the editor and yourself for providing feedback on our article entitled "Efficacy of OLIF combined with pedicle screw internal fixation for lumbar spinal stenosis on spinal canal changes before and after surgery", which was recently published in the Journal of Orthopedic Surgery and Research. We are grateful for your keen interest in our research and the insightful points you raised. In this letter, we aim to address and diskuss these matters in detail:

1. Heterogeneous patient population: the study population in their research was heterogeneous, with different lumbar levels and pathologies. This can make it challenging to draw definitive conclusions about the effectiveness of the procedure for specific subgroups of patients.
  Heterogeneous patient population: We acknowledge the heterogeneity of the patient population in our study [1], which included individuals with different lumbar levels and pathologies. This heterogeneity could potentially limit the generalizability of our findings to specific subgroups of patients. We have diskussed this limitation in **the fourth paragraph of the diskussion section, lines 35–45, where we stated, **“*It is important to acknowledge that the small sample size in this study may limit the reliability of the results. Additionally, the absence of specifc surgical segments and individual variations among patients could potentially afect result consistency. Furthermore, the followup observation was limited to a duration of only 1 year, while longer-term tracking could ofer more compelling evidence. Terefore, further validation through larger sample sizes and comprehensive analysis of core data from multiple perspectives is still necessary.*” We have emphasized the need for further research to address these limitations.
2. Small sample sizes: small sample sizes can limit the generalizability of the findings. Larger studies with more participants are needed to provide more robust evidence.
  Small sample sizes: We appreciate your point regarding the small sample sizes in our study. We agree that larger studies with more participants are needed to provide more robust evidence and enhance the generalizability of the findings. We have acknowledged this limitation in the aforementioned paragraph and emphasized the need for further validation through larger sample sizes.
3. Short follow-up duration: Longer-term follow-up is necessary to assess the durability and long-term outcomes of the procedure.
  Short follow-up duration: We acknowledge that the follow-up duration in our study was limited to one year, which may not fully capture the long-term outcomes and durability of the procedure. We have addressed this limitation in the same paragraph mentioned above, where we highlighted the importance of longer-term tracking to provide more compelling evidence. We agree that future studies should include extended follow-up periods to evaluate the long-term outcomes of OLIF combined with pedicle screw internal fixation for lumbar spinal stenosis.
4. Lack of comparison groups: there was no direct comparison between OLIF combined with pedicle screw internal fixation and other treatment approaches or control groups. Comparative studies are essential to determine the relative efficacy and safety of this procedure compared to alternative treatments.
  We fully agree with the importance Dr. Alimohammadi highlighted regarding the lack of a control group, and we sincerely appreciate this valuable suggestion. In fact, during the design of this study, we carefully considered whether to include traditional posterior lumbar interbody fusion (PLIF) or minimally invasive transforaminal lumbar interbody fusion (MIS-TILF) as a control group for our research. However, after an extensive review of the relevant literature, we found that comparative studies of this nature primarily focused on clinical parameters such as intraoperative blood loss, operative time, and postoperative weight-bearing time. Even when radiological parameters were considered, the analysis was limited to superficial comparisons of parameters such as intervertebral height, foraminal height, and lumbar lordotic angle [2–4].
  If we were to compare one of these surgical approaches with oblique lumbar interbody fusion (OLIF) combined with pedicle screw fixation for the treatment of lumbar spinal stenosis (LSS), we believe it would replicate previous studies and lack innovation. Therefore, after careful consideration, we decided to focus the study on radiological parameters. We specifically described the changes in parameters such as disk height (DH), cross-sectional area of the vertebral canal (CSAVC), cross-sectional area of the dural sac (CSADS), cross-sectional area of the intervertebral foramen (CSAIF), spinal canal volume (SCV), and the expansion rate of SCV in patients undergoing OLIF combined with pedicle screw fixation for LSS. These parameters were used to evaluate the indirect decompression effect of OLIF combined with pedicle screw fixation.
  Additionally, we presented line graphs depicting the changes in disk height (DH) and cross-sectional area of the vertebral canal (CSAVC) before surgery, after surgery, and one year postoperatively (Fig. 1). These graphs were used to support the intervention effects of OLIF combined with pedicle screw fixation for LSS. Furthermore, we conducted Pearson correlation analyses between the increase in disk height (DH) and the expansion rate of SCV and the decrease in Oswestry Disability Index (ODI) (Fig. 2). These analyses aimed to explore the correlation between radiological parameters and functional outcomes, further evaluating the effectiveness and safety of OLIF combined with pedicle screw fixation for LSS. By focusing on these radiological parameters and their correlation with functional outcomes, we aimed to provide a comprehensive evaluation of the effectiveness and safety of OLIF combined with pedicle screw fixation for LSS. We believe that this approach adds value to the existing literature and contributes to the understanding of this surgical technique in the context of lumbar spinal stenosis treatment.

**Fig. 1:**
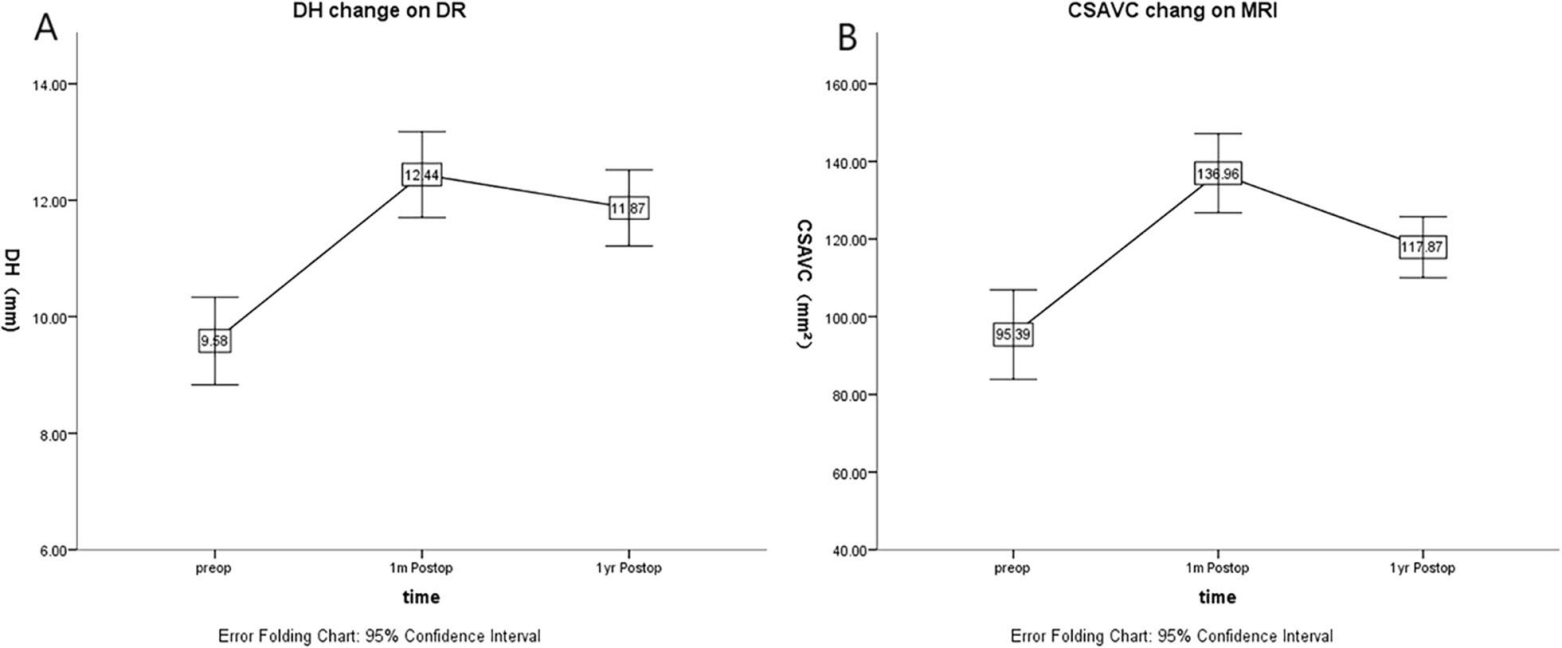
DH (the disk height) change on DR and CSAVC (the cross-sectional area of vertebral canal) change on MRI. **A** A plot of significant change in height on DR preoperative, 1 month postoperative, and 1 year postoperative after DH and **B** a plot of significant change in the area on MRI preoperative, 1 month postoperative, and 1 year postoperative after CSAVC

**Fig. 2:**
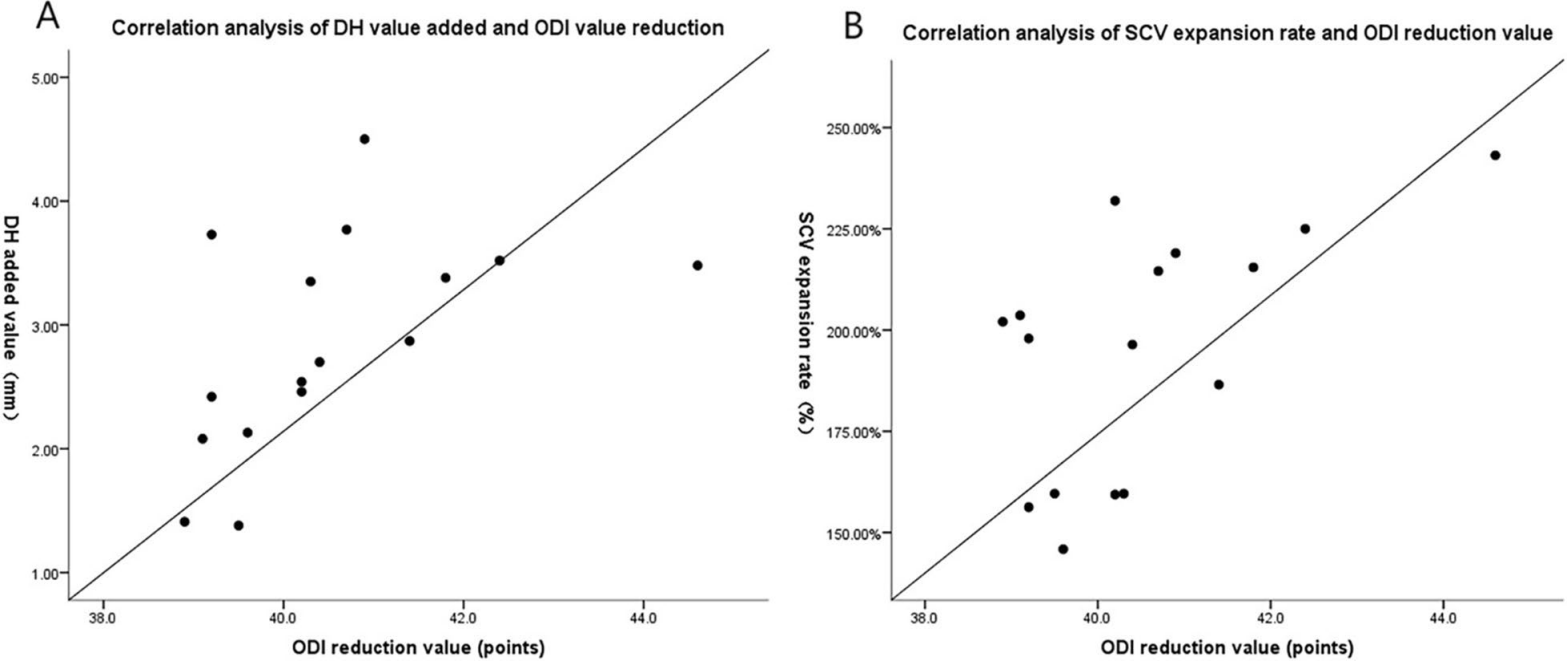
Person correlation analysis of the increase of DH and the expansion of SCV and the decrease of ODI, respectively. **A** The correlation analysis between the increased value of DH and the reduced value of ODI as shown, with a significant correlation (*r *= 0.535, *p *= 0.033) and **B** the correlation analysis between the expansion rate of SCV and the reduced value of ODI as shown, with a significant correlation (*r *= 0.586, *p *= 0.017), *p *< 0.05
  Finally, Throughout the main text, we also emphasized the differences between our study and previous research. For example, **in the Introduction section, lines 26–45, we state**, “*At present, most clinical studies comparing treatments for LSS focus on comparing OLIF and MI-TILF. However, these studies mainly focus on clinical parameters such as intraoperative blood loss, operative time, and hospitalization days. Even if they do analyse radiological parameters, it is only a superficial analysis of sagittal sequence parameters such as disk height and lumbar lordotic angle. There is a lack of research on the changes in the spinal canal before and after OLIF combined with pedicle screw fixation, which can reflect the indirect decompression effect of LSS. Therefore, the purpose of this study is to compare the changes in disk height (DH), cross-sectional area of the vertebral canal (CSAVC), cross-sectional area of the spinal canal (CSADC), crosssectional area of the intervertebral foramen (CSAIF), spinal canal volume (SCV), and SCV expansion rate before and after OLIF combined with pedicle screw fixation in LSS patients.*” **and in the diskussion section, lines 1–35, we state,**“*The previous studies have primarily focused on single retrospective studies of oblique lumbar interbody fusion (OLIF) treatment for lumbar spinal stenosis (LSS), comparative studies between OLIF and other surgical methods, or diagnostic analysis through CT or MRI measurements of the dura mater. While existing studies have underscored the beneficial impact of OLIF surgery in treating patients with LSS, their primary focus has been on clinical parameters such as intraoperative blood loss, duration of surgery, and time taken to ambulate post-surgery. Even when radiological parameters were scrutinized, the analysis was largely limited to a cursory examination of sagittal sequence parameters such as disk height and lumbar lordotic angle. However, there remains a significant gap in the current research landscape concerning the changes in the spinal canal both pre- and post-surgery in patients with lumbar spinal stenosis (LSS) who have undergone oblique lumbar interbody fusion (OLIF) in combination with pedicle screw fixation. This is the crux of our study—to evaluate the indirect decompression effect of OLIF combined with pedicle screw fixation in treating LSS, as evidenced by changes in the spinal canal. In this study, we aimed to assess the indirect decompression effect of OLIF combined with pedicle screw internal fixation on LSS patients by comparing various parameters such as disk height (DH), cross-sectional area of the vertebral canal (CSAVC), cross-sectional area of the dural sac (CSADS), cross-sectional area of the intervertebral foramen (CSAIF), and spinal canal volume (SCV), as well as the SCV expansion rate. By analysing these parameters, we aimed to provide more data and explicit evidence regarding the indirect decompression achieved through OLIF combined with pedicle screw internal fixation.*”
5. Selection bias: there may have been selection bias in their patient cohorts, which can affect the generalizability of the results.
  We acknowledge the issue of patient selection bias mentioned above. In our study, we employed specific inclusion criteria to select participants, which may have resulted in some degree of selection bias. We made efforts to ensure the rationality and accuracy of the inclusion criteria to minimize the impact of this bias. However, we also recognize that the selection of inclusion criteria is limited and may not fully encompass all potential patient populations. Future research can further broaden the scope of inclusion criteria to more comprehensively represent the patient population. We humbly acknowledge this limitation and strive to address it in future studies. We appreciate your reminder regarding this issue and commit to addressing it in future research to enhance the reliability and applicability of our findings.
6. In your letter, you provided valuable insights from another study comparing standalone OLIF with OLIF combined with posterior bilateral percutaneous pedicle screw fixation for the treatment of lumbar spondylolisthesis [5]. The study highlighted differences in clinical outcomes, cage subsidence rates, fusion rates, and the relationship between cage subsidence and adjacent vertebral fractures. These findings contribute to our understanding of the benefits and considerations associated with different surgical approaches. Furthermore, you mentioned a study by Liu et al. [6] comparing OLIF and minimally invasive transforaminal lumbar interbody fusion (MISTLIF) for the treatment of degenerative lumbar spinal stenosis (DLSS). The study revealed advantages of OLIF in terms of reduced surgical invasion, lower incidence of postoperative low back pain, faster recovery, and decreased anxiety levels compared to MISTLIF. However, it is important to consider the higher cost and longer operative time associated with OLIF.
  Overall, we appreciate your thoughtful diskussion and the additional insights you provided. The studies you mentioned contribute to the growing body of evidence regarding the efficacy and outcomes of different surgical approaches for lumbar spinal stenosis. We encourage further research in this field to enhance our understanding and guide clinical decision-making.

Thank you for your valuable contribution to the scientific diskourse.

## Data Availability

All methods were carried out in accordance with relevant guidelines and regulations.
